# A core of kinase-regulated interactomes defines the neoplastic MDSC lineage

**DOI:** 10.18632/oncotarget.4746

**Published:** 2015-07-23

**Authors:** Maria Gato-Cañas, Xabier Martinez de Morentin, Idoia Blanco-Luquin, Joaquin Fernandez-Irigoyen, Isabel Zudaire, Therese Liechtenstein, Hugo Arasanz, Teresa Lozano, Noelia Casares, Apirat Chaikuad, Stefan Knapp, David Guerrero-Setas, David Escors, Grazyna Kochan, Enrique Santamaría

**Affiliations:** ^1^ Immunomodulation group, Navarrabiomed-FMS, IdiSNA, Pamplona, Spain; ^2^ Immunomodulation group, Division of Infection and Immunity, University College London, UK; ^3^ Proteomics Unit, Navarrabiomed-FMS, Proteored-ISCIII IdiSNA, Pamplona, Spain; ^4^ Hospital de Navarra, Department of Oncology, IdiSNA, Pamplona, Spain; ^5^ Immunology and Immunotherapy Program, Center for Applied Medical Research, University of Navarra, IdiSNA, Pamplona, Spain; ^6^ Structural Genomics Consortium (SGC), University of Oxford, Headington, UK; ^7^ Institute for Pharmaceutical Chemistry, Johann Wolfgang Goethe-University, Frankfurt, Germany; ^8^ Cancer Epigenetics group, Navarrabiomed-FMS, IdiSNA, Pamplona, Spain; ^9^ Protein Production Unit, Navarrabiomed-FMS, IdiSNA, Pamplona, Spain

**Keywords:** MDSC, proteomics, interactomes, kinases, therapeutic targets

## Abstract

Myeloid-derived suppressor cells (MDSCs) differentiate from bone marrow precursors, expand in cancer-bearing hosts and accelerate tumor progression. MDSCs have become attractive therapeutic targets, as their elimination strongly enhances anti-neoplastic treatments. Here, immature myeloid dendritic cells (DCs), MDSCs modeling tumor-infiltrating subsets or modeling non-cancerous (NC)-MDSCs were compared by in-depth quantitative proteomics. We found that neoplastic MDSCs differentially expressed a core of kinases which controlled lineage-specific (PI3K-AKT and SRC kinases) and cancer-induced (ERK and PKC kinases) protein interaction networks (interactomes). These kinases contributed to some extent to myeloid differentiation. However, only AKT and ERK specifically drove MDSC differentiation from myeloid precursors. Interfering with AKT and ERK with selective small molecule inhibitors or shRNAs selectively hampered MDSC differentiation and viability. Thus, we provide compelling evidence that MDSCs constitute a distinct myeloid lineage distinguished by a “kinase signature” and well-defined interactomes. Our results define new opportunities for the development of anti-cancer treatments targeting these tumor-promoting immune cells.

## INTRODUCTION

Anti-cancer treatments are primarily aimed at causing arrest of tumor cell growth or tumor cell death. In recent years, immunotherapy has resurfaced as an attractive therapeutic alternative [1]. However, the expansion of immunosuppressive cell types in cancer patients strongly interferes with anti-tumor immune responses. These immunosuppressive cells enhance tumor progression/metastasis and counteract classical anti-neoplastic treatments. Amongst these, myeloid-derived suppressor cells (MDSCs) are major contributors to tumor progression. MDSCs differentiate from precursors within the bone marrow (BM) in tumor-bearing hosts. MDSCs distribute systemically and infiltrate tumors, where they contribute to tumor progression through a variety of mechanisms [2, 3]. However, MDSC differentiation and functions are still poorly understood. This is due to the difficulty of isolating them from tumor-bearing subjects, or differentiating them *in vitro* so that they faithfully model *in vivo* cell subsets [4]. Nonetheless, counteracting their activities strongly enhances anti-cancer treatments [5]. Thus, finding treatments that would specifically eliminate MDSCs could improve the efficacy of anti-cancer therapies.

While the most valuable source of MDSCs for research is the tumor itself, their isolation is still a challenge [4, 6]. Therefore, other sources such as spleen or blood are widely used. However, these MDSCs are phenotypically and functionally different from tumor-infiltrating subsets [6–9]. To overcome these difficulties, we developed an *ex vivo* differentiation system that produces MDSCs modeling tumor-infiltrating subsets (B16-MDSCs) and non-cancerous (NC) MDSCs (293T-MDSCs) [8]. These *ex vivo* MDSCs have been phenotypically and functionally validated in B16 melanoma and CT26 colorectal cancer models [8–11].

The use of high-throughput analytical techniques for the identification of cellular regulatory pathways and novel molecular targets is on the increase. Two independent studies on the proteome of blood and spleen MDSCs have been published using LC-MS/MS mass spectrometry and label-free quantification [12, 13]. Although relevant data was obtained, none of these studies included control cell types such as myeloid DCs and NC-MDSCs. Therefore, studies that have been published so far have not discriminated pathways associated to cell lineage or the tumor environment.

To overcome these issues, we carried out in-depth proteomic analyses comparing myeloid DCs, MDSCs modeling tumor-infiltrating subsets or modeling NC-MDSCs. We found a kinase signature that defined neoplastic MDSCs which could be specifically targeted to interfere with MDSC differentiation from myeloid precursors.

## RESULTS

### MDSC lineage-specific interactomes

**Figure 1:**
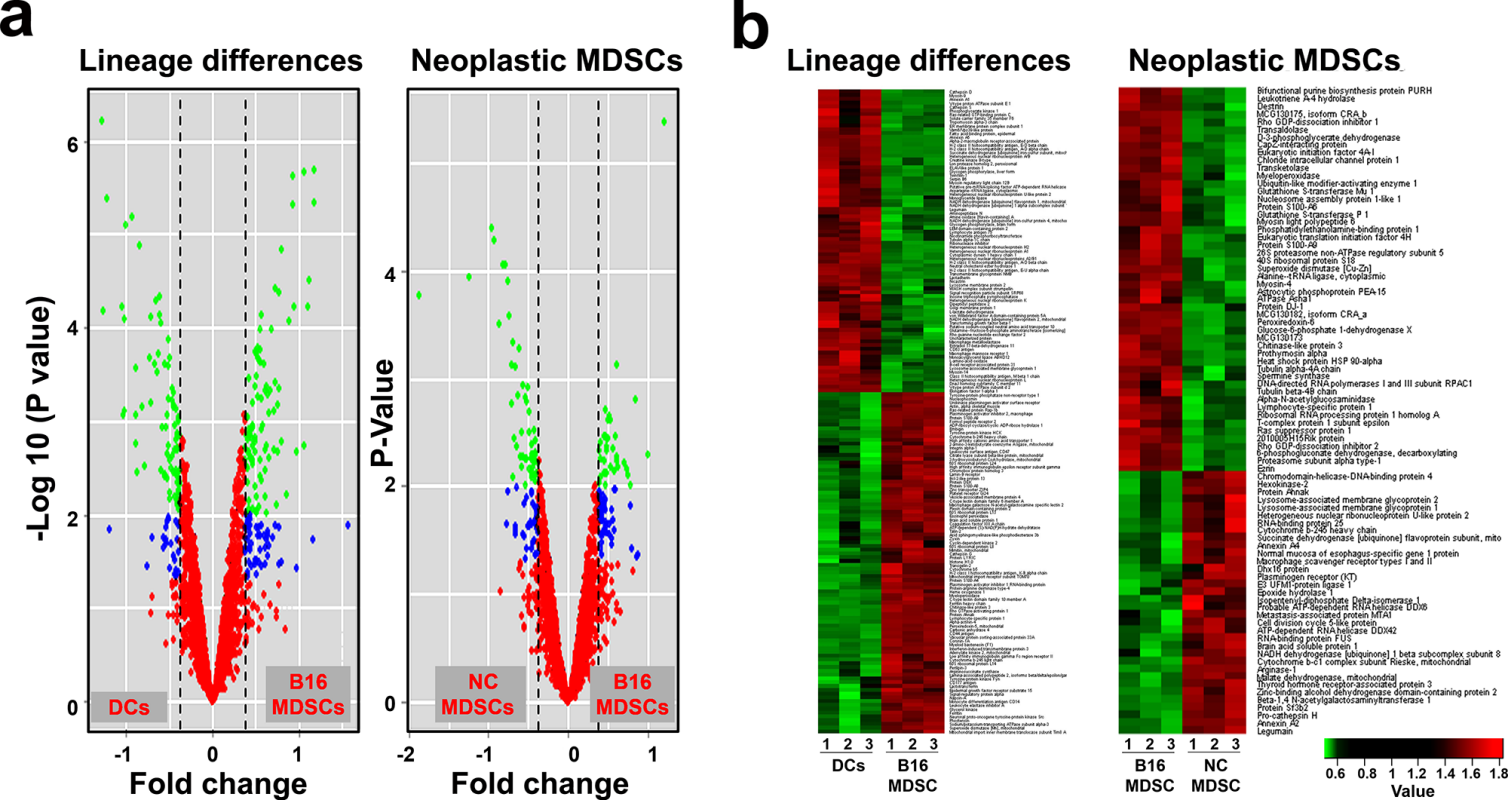
Differentially expressed proteins in MDSCs caused by lineage and cancer **a.** Volcano plots representing the fold-change of identified proteins with associated *P* values from the pair-wise quantitative comparisons of DCs vs B16-MDSCs (lineage differences, left plot) and NC-MDSCs vs B16-MDSCs (cancer-regulated differences, right plot). In green, very significantly changed proteins (*P* < 0.01), in blue, significantly changed proteins (*P* < 0.05) and in red, unchanged proteins between the pair-wise comparisons. **b.** Heat map representing the degree of change for the differentially expressed proteins (*P* < 0.01, Supplementary Table 1) between the indicated samples (independent biological triplicates are indicated as 1, 2 and 3; DCs, dendritic cells; B16-MDSCs, cancerous MDSCs; NC-MDSCs, non-cancerous MDSCs), as shown below. Legend (bottom right) indicates color-coded fold-change on Log_10_ scale. Red and green, up- and down-regulated proteins, respectively.

**Figure 2:**
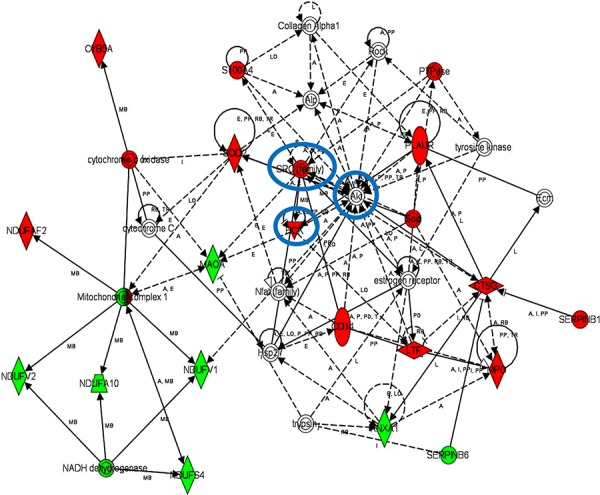
Functional MDSC lineage-specific interactome networks controlled by SRC, HCK and AKT kinases Graph represents functional interactomes constructed with Ingenuity Pathway Analysis tool using lineage specific (B16-MDSCs vs DCs) differentially expressed proteins, which shows detailed interaction relationships between the input nodes (differentially expressed proteins between MDSCs and DCs), and regulatory kinases encircled in blue. This interactome links AKT/SRC kinases with mitochondrial respiration and dysfunction, protection against oxidative stress and extracellular matrix remodeling. Nodes in red, up-regulated proteins. Nodes in green, down-modulated proteins. In white, predicted protein nodes. A, activation; B, binding; C, causes/leads to; CC, chemical-chemical interaction; CP, chemical-protein interaction; E, expression; EC, enzyme catalysis; I, inhibition; L, proteolysis; LO, localization; M, biochemical modification; MB, group/complex; P, phosphorylation/dephosphorylation; PD Protein-DNA binding; PP Protein-Protein binding; PR Protein-RNA binding; RB Regulation of Binding; RE Reaction; RR RNA-RNA Binding; T Transcription; TR Translocation. Dash arrows, indirect interactions.

**Figure 3:**
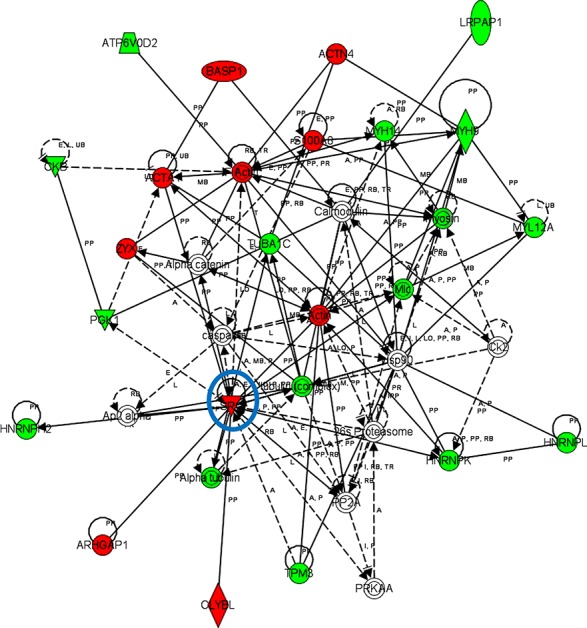
Functional MDSC lineage-specific interactome networks regulating cytoskeletal changes and controlled by SRC kinases Graph presents functional interactomes constructed with Ingenuity Pathway Analysis tool using lineage specific (B16-MDSCs vs DCs) differentially expressed proteins, which shows detailed interaction relationships between the input nodes (differentially expressed proteins between MDSCs and DCs), and regulatory kinases encircled in blue. This interactome links SRC kinases with protein transport, mRNA processing, cytoskeletal re-organization and decreased glycolysis. Nodes in red, up-regulated proteins. Nodes in green, down-modulated proteins. In white, predicted protein nodes. A, activation; B, binding; C, causes/leads to; CC, chemical-chemical interaction; CP, chemical-protein interaction; E, expression; EC, enzyme catalysis; I, inhibition; L, proteolysis; LO, localization; M, biochemical modification; MB, group/complex; P, phosphorylation/dephosphorylation; PD Protein-DNA binding; PP Protein-Protein binding; PR Protein-RNA binding; RB Regulation of Binding; RE Reaction; RR RNA-RNA Binding; T Transcription; TR Translocation. Dash arrows, indirect interactions.

**Figure 4:**
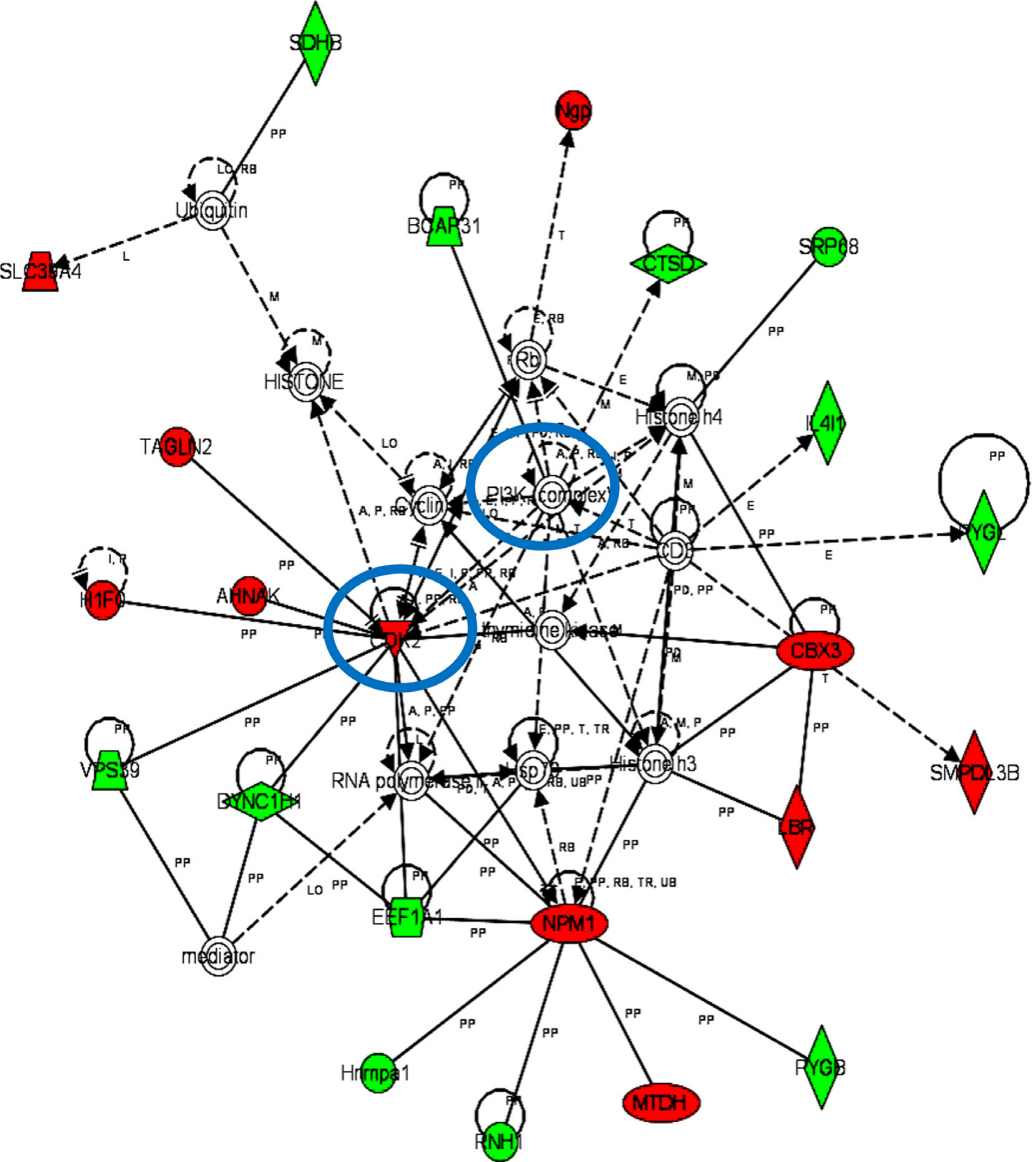
Functional MDSC lineage-specific interactome networks controlled by PI3K and CDK2 kinases Graph presents functional interactomes constructed with Ingenuity Pathway Analysis tool using lineage specific (B16-MDSCs vs DCs) differentially expressed proteins, which shows detailed interaction relationships between the input nodes (differentially expressed proteins between MDSCs and DCs), and regulatory kinases encircled in blue. This interactome links PI3K with cell cycle, protein synthesis and transport, survival and proliferation. Nodes in red, up-regulated proteins. Nodes in green, down-modulated proteins. In white, predicted protein nodes. A, activation; B, binding; C, causes/leads to; CC, chemical-chemical interaction; CP, chemical-protein interaction; E, expression; EC, enzyme catalysis; I, inhibition; L, proteolysis; LO, localization; M, biochemical modification; MB, group/complex; P, phosphorylation/dephosphorylation; PD Protein-DNA binding; PP Protein-Protein binding; PR Protein-RNA binding; RB Regulation of Binding; RE Reaction; RR RNA-RNA Binding; T Transcription; TR Translocation. Dash arrows, indirect interactions.

iTRAQ-based quantitative proteomics were performed on MDSCs modeling melanoma-infiltrating subsets (B16-MDSCs), using immature myeloid DC proteomes as a comparative standard to identify melanoma MDSC lineage-specific interactomes. 3609 proteins were unambiguously identified with an FDR lower than 1%. Differential protein quantification was performed between DCs and B16-MDSCs, and the most affected proteins with a significance level of 0.01 were used for further analyses (Fig. 1a). Expression of 58 proteins was found up-regulated in MDSCs while 46 were down-modulated (Fig. 1b and Supplementary Table 1). Ingenuity Pathway Analysis was used to reconstruct functional interactome maps with differentially expressed proteins. Three distinct interactomes resulted from the analyses, with highly detailed interaction relationships between nodes (Figs. 2, 3, 4). The top canonical pathways which separated B16-MDSCs from DCs were: (1) mitochondrial dysfunction (*P* = 1.5 × 10^−7^); (2) leukocyte extravasation signaling (*P* = 5 × 10^−6^), (3) caveolar-mediated endocytosis signaling (*P* = 2.6 × 10^−5^) and (4) integrin signaling (4 × 10^−5^). These pathways were associated to SRC, FYN and HCK kinases, unambiguously identified by mass spectrometry (Supplementary Table 1). Protein interactome networks predicted a number of regulatory proteins (hubs) including the PI3K-AKT signaling axis (Fig. 2). Importantly, SRC kinases controlled changes in the cytoskeleton and mitochondrial dysfunction through down-regulation of complex I NAPDH dehydrogenase subunits (Figs. 2, 3). These kinases were directly associated to various molecular nodes such as calmodulin, Hsp90, α-catenin and the proteasome (Fig. 4).

**Table 1:** IC50s of small molecule inhibitors over myeloid precursors committed towards DC or MDSC differentiation

Inhibitor	IC50, DCs	IC50, MDSC	Targeted kinases
**AKT inhibitor X**	>100 μM	3.9 ± 0.6 μM	AKT
**TX1123**	3.2 ± 1.4 μM	3.4 ± 3 μM	SRC eEF2-K PKA
**Saracatinib**	3.5 ± 3.4 μM	8.8 ± 8 μM	SRC FYN
**PP2**	46.5 ± 0.7 μM	45.4 ± 2 μM	FYN HCK
**PD0325901**	44.7 ± 4.5 nM	6.2 ± 2.8 nM	MEK
**SCH772984**	86.5 ± 19 nM	21 ± 15 nM	ERK1
**VTX-11e**	8 ± 0.15 μM	1.3 ± 0.14 μM	ERK1
**Gö 6983**	5.7 ± 1 μM	5.7 ± 2.3 μM	PKCα, β, γ, δ, ζ and μ
**NPC-15437**	8 ± 2.3 μM	8 ± 2 μM	PKC

Confidence-based protein networks were reconstructed using STRING software [14], with up-regulated or down-regulated proteins. Both high and medium confidence links were considered (score >0.4), as the number of networks was limited to allow careful confirmation. About 10 distinct protein networks were organized around a central group of kinases that included SRC family members (Supplementary Fig. 1). These networks were associated to production of reactive oxygen species (ROS), protection against oxidative damage, intracellular vesicle trafficking and aminoacid metabolism. Decreases in spliceseosomal proteins, carbohydrate metabolism, lysosomal function and MHC II antigen presentation were also evident.

**Figure 5:**
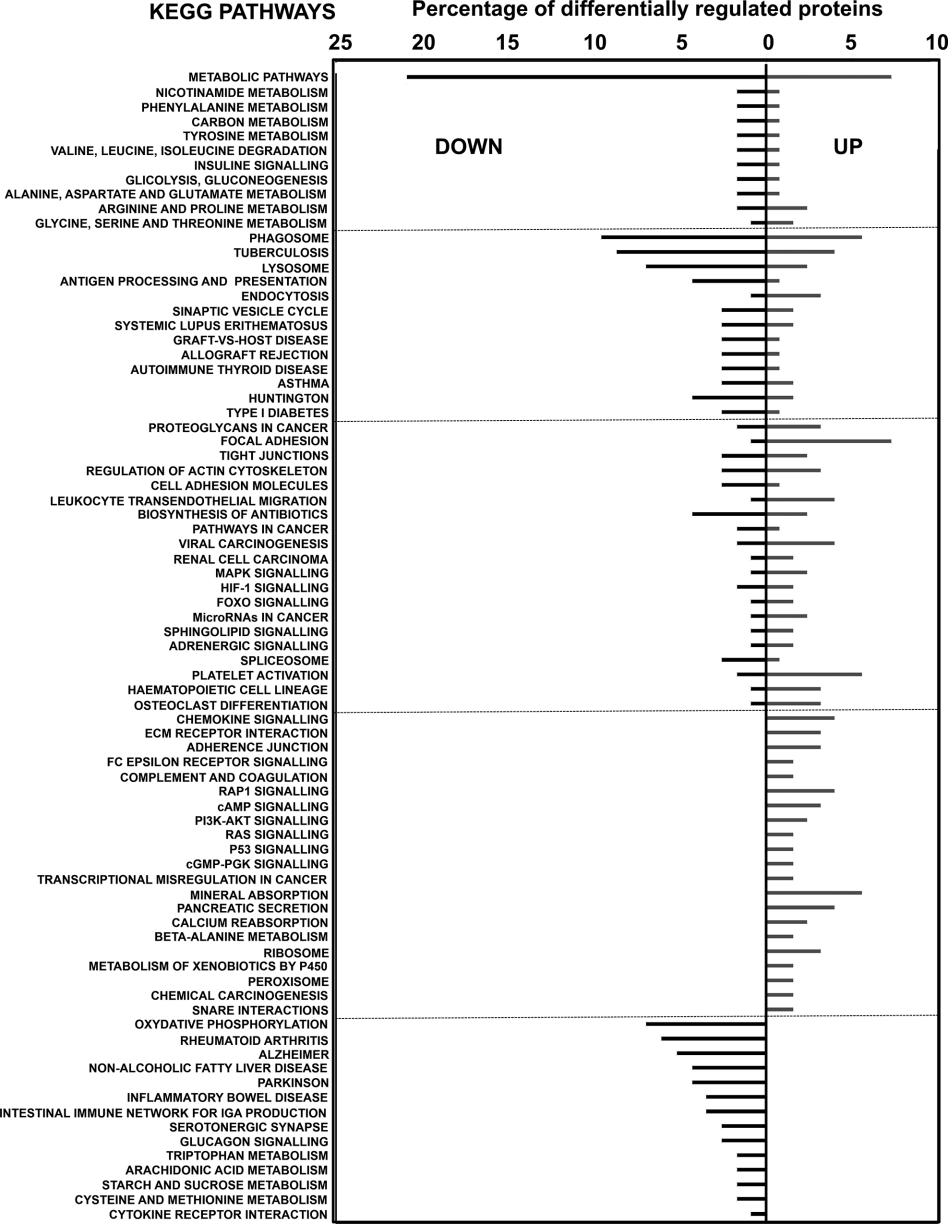
KEGG pathway analyses of differentially-expressed proteins in MDSCs compared to myeloid DCs Graph represents the percentage of differentially up- or down-regulated (as indicated within the graph) proteins ascribed to the indicated KEGG pathways.

KEGG pathway mapping was applied to up- and down-regulated proteins. KEGG analyses showed strong inhibition of cellular processes associated to inflammatory disorders and a decrease in metabolism of aminoacids (Fig. 5).

### Cancer-specific interactomes in MDSCs modeling tumor-infiltrating subsets

**Figure 6:**
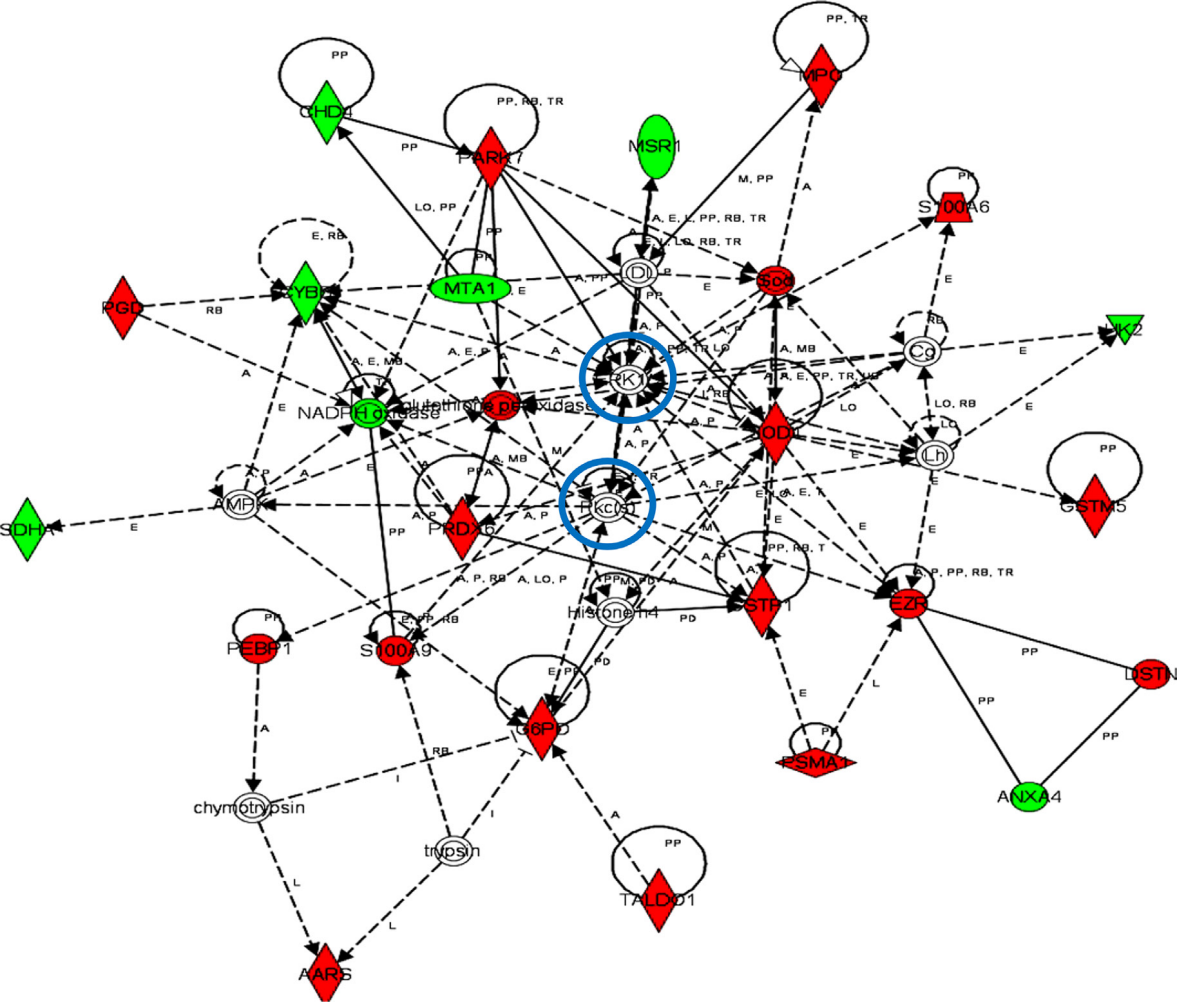
Functional interactomes with cancer-regulated (B16-MDSCs vs NC-MDSCs) differentially expressed proteins Ingenuity Analysis interactome linking ERK and PKCs with protection against oxidative stress, mitochondrial electron transport and NADPH oxidase activity, the pentose phosphate pathway and ROS generation. Regulatory kinases are encircled in blue. Nodes in red, up-regulated proteins. Nodes in green, down-modulated proteins. In white, predicted protein nodes; A, activation; B, binding; C, causes/leads to; CC, chemical-chemical interaction; CP, chemical-protein interaction; E, expression; EC, enzyme catalysis; I, inhibition; L, proteolysis; LO, localization; M, biochemical modification; MB, group/complex; P, phosphorylation/dephosphorylation; PD Protein-DNA binding; PP Protein-Protein binding; PR Protein-RNA binding; RB Regulation of Binding; RE Reaction; RR RNA-RNA Binding; T Transcription; TR Translocation. Dash arrows, indirect interactions.

Our *ex vivo* system generates MDSCs that model tumor-infiltrating (B16-MDSCs) and non-cancerous NC-MDSCs (293T-MDSCs) [8, 9, 11]. It has to be pointed out that NC-MDSCs are not precursors of tumor-infiltrating MDSCs, but cells differentiated *ex vivo* in non-neoplastic conditions as described [8, 9]. Thus, a quantitative proteomic comparison between these two subsets was performed to highlight cancer-regulated pathways. These analyses uncovered 50 up- and 35 down-regulated proteins in B16-MDSCs compared to NC-MDSCs, and pathway reconstruction was performed using Ingenuity (Fig. 6, Supplementary Table 1). The top canonical pathways which differentiated neoplastic from non-cancerous MDSCs were: (1) the pentose phosphate pathway (*P* = 6.4 × 10^−8^), represented by G6PD, PGD and TALDO1 up-regulation; (2) epithelial adherence junction signaling with up-regulation of EZR, DSTN, tubulin and Rho-like proteins (*P* = 2.4 × 10^−3^). The two top associated molecular and cellular functions were (1) free radical scavenging and oxidative stress responses (*P* = 1.1 × 10^−8^) as indicated by up-regulation of SOD2, MPO, PRDX, GSTM5 and PARK7 amongst others, and (2) carbohydrate metabolism which was associated to the pentose phosphate pathway (*P* = 2.5 × 10^−8^). Interestingly, Ingenuity protein interaction networks included the kinases ERK1 and PKC isoforms as regulatory hubs (Fig. 6).

**Figure 7:**
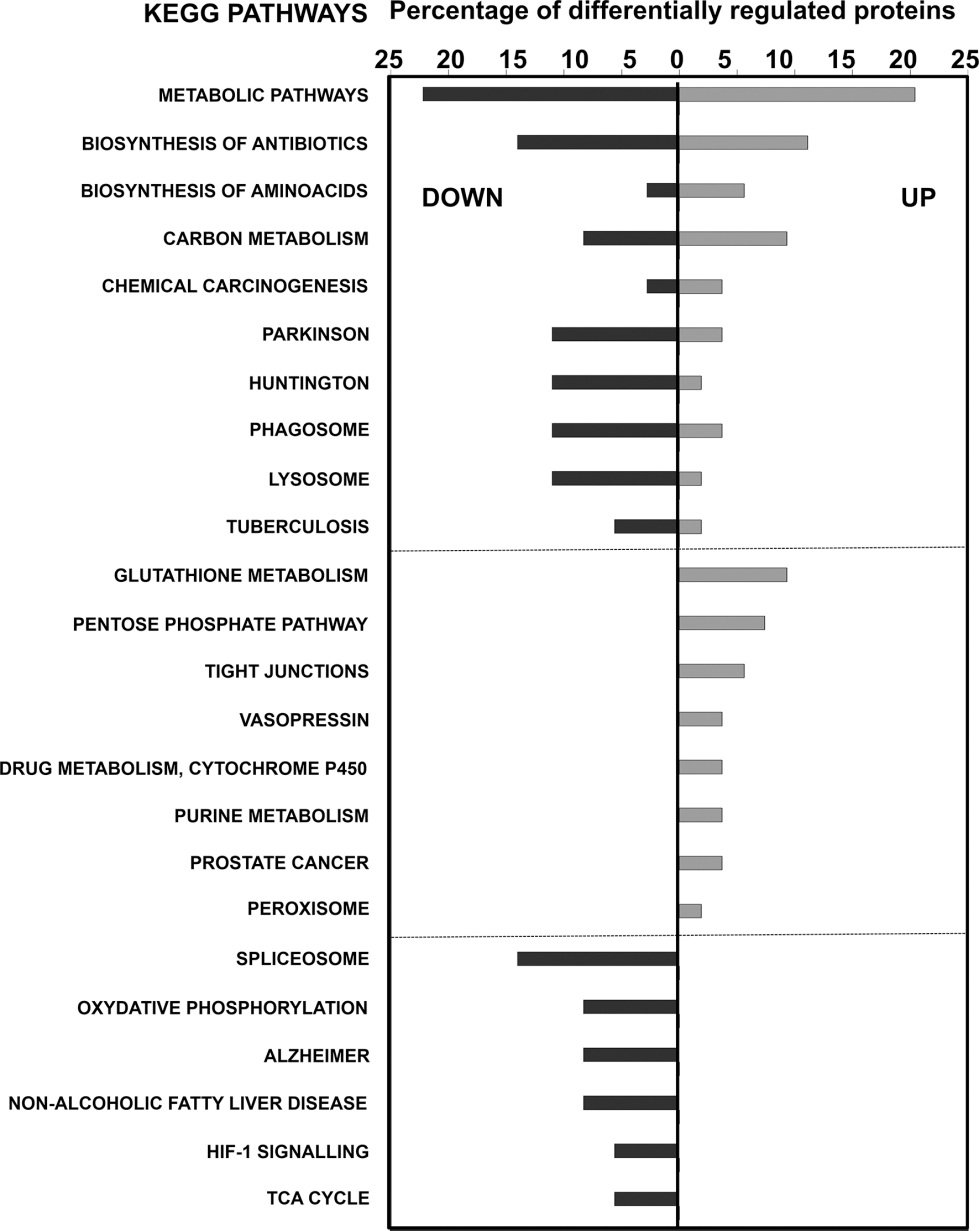
KEGG pathway analyses of differentially-expressed proteins in MDSCs modeling tumor-infiltrating subsets compared to NC-MDSCs The graph shows the percentage of differentially up- or down-regulated (as indicated within the graph) proteins ascribed to the indicated KEGG pathways.

Similar results were obtained with STRING software (Supplementary Fig. 2). Most notably, up-regulation of the pentose phosphate pathway, changes in cytoskeletal proteins and down-modulation of oxidative phosphorylation. Results from KEGG pathway mapping highlighted increased glutathione metabolism, activation of the pentose phosphate pathway and a decrease in spliceosomal proteins (Fig. 7).

### A kinase signature defines the neoplastic MDSC lineage

**Figure 8:**
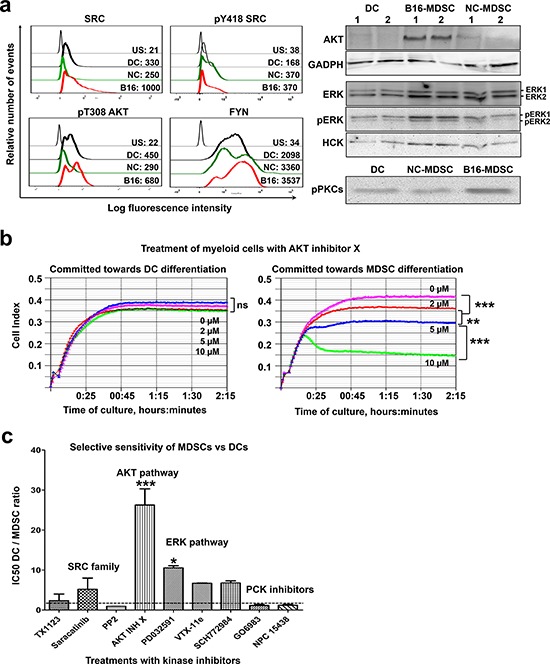
A kinase signature discriminates MDSCs from DCs **a.** Graphs on the left, flow cytometry histograms with expression profiles of the indicated kinases in DCs, NC-MDSCs and B16-MDSCs. Mean fluorescent intensities for each cell population are shown within the graphs. US, unstained control; DC, dendritic cells; NC, non-cancerous MDSCs; B16, MDSCs modeling tumor-infiltrating subsets. Blots on the right, detection of the indicated kinases by immunoblotting. Two preparations from the indicated cell populations (top of the immunoblots) were loaded and lanes were labelled as 1 and 2. An immunoblot for GADPH detection is shown as a reference control, on the same membrane used for AKT detection above. **b.** Representative real-time cell monitoring (RTCA) results for myeloid precursors treated with the indicated concentrations of AKT inhibitor X, and grown either in DC-differentiation medium or in B16-MDSC conditioning medium as indicated on top. Data is plotted as means of cell index with error bars (standard deviations) from duplicate cultures, shown as a function of time. Relevant statistical comparisons are shown and indicated with *, **, and *** for significant (*P* < 0.05), very significant (*P* < 0.01) and highly significant (*P* < 0.001) differences, respectively. **c.** DC/MDSC IC50 ratios calculated for the indicated treatments. Ratios close to 1 (horizontal dotted line) indicate that treatments are equally inhibitory over MDSCs and DCs. Ratios higher than 1 indicate that MDSCs are more sensitive to the specific treatments than DCs.

Systems biology analyses delineated a kinase signature of the MDSC lineage (AKT and the SRC family, which included SRC, HCK and FYN) and neoplastic MDSCs (ERK and PKC kinases). Overall, the expression of FYN, HCK and total and phosphorylated SRC agreed with proteomic data, as assessed by flow cytometry and immunoblotting. The predicted participation of AKT was also confirmed (Fig. 8a). AKT expression was particularly high in MDSCs modeling tumor-infiltrating subsets as detected by immunoblotting. ERK1 and PKC isoforms were predicted to be differentially expressed in tumor-infiltrating MDSCs. While total ERK expression was equivalent between B16-MDSCs and NC-MDSCs, phosphorylated (active) ERK1 was increased in B16-MDSCs (Fig. 8a). The expression of phosphorylated PKC isoforms (phosphorylated pan-PKCs) was tested by immunoblot. In agreement with proteomic data and Ingenuity analyses, phosphorylated PKCs were present at higher levels in MDSCs modeling neoplastic subsets (Fig. 8a).

### AKT and ERK1 specifically contribute to MDSC differentiation from myeloid precursors

**Figure 9:**
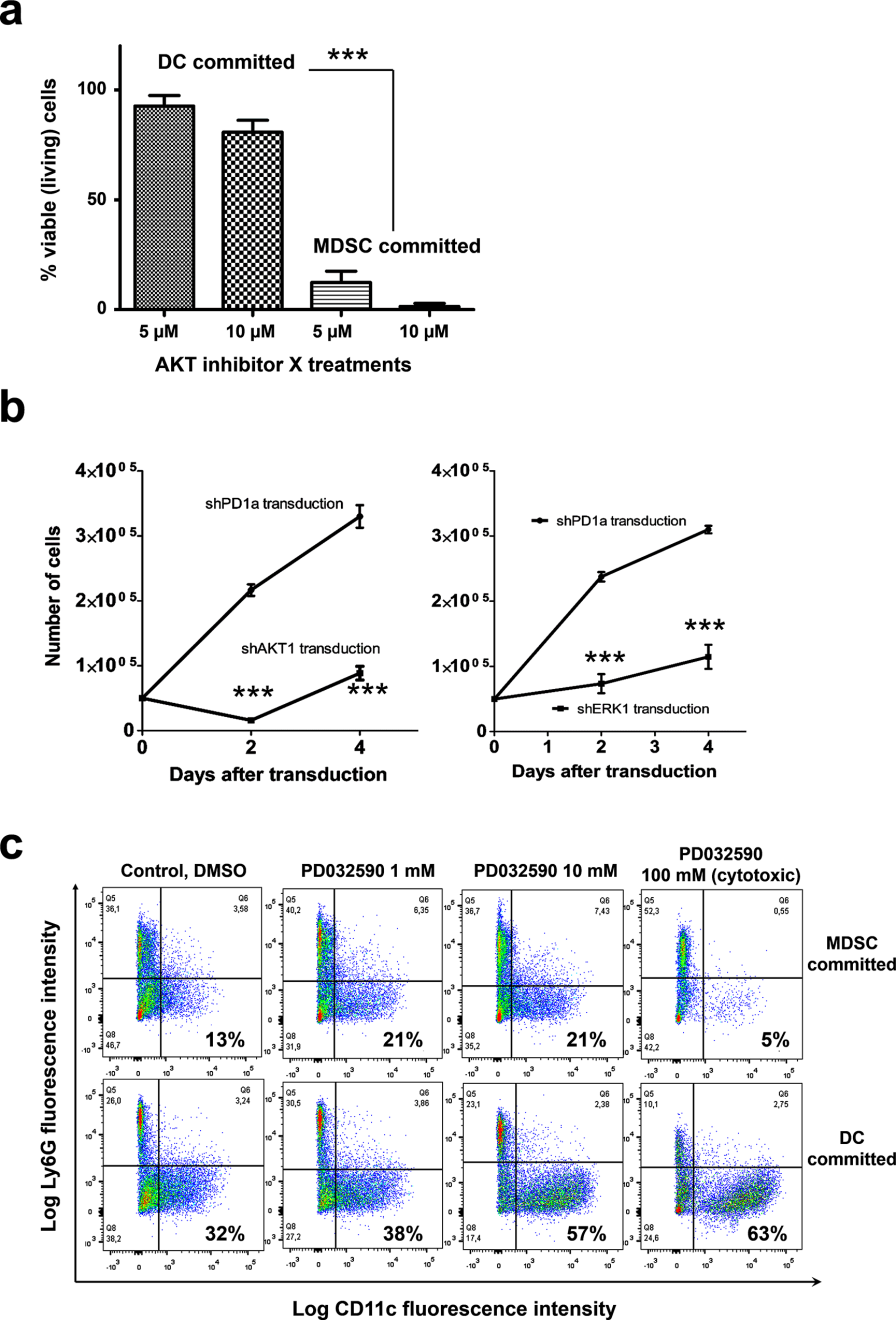
AKT is required for survival of myeloid cells committed to MDSC differentiation, while inhibition of the ERK pathway enhances conventional DC differentiation **a.** The percentages of viable myeloid precursors treated with the indicated concentrations of AKT inhibitor X are indicated as a bar graph with standard deviations as error bars. Precursors were committed towards DC or MDSC differentiation as indicated on top of the bars. Viable cells were quantified following trypan blue staining. Relevant statistical comparisons are shown. **b.** Graph on the left, growth of myeloid cell precursors transduced with a lentivector encoding a control shRNA (shPD1a), or an AKT1-specific shRNA (shAKT1). Data is plotted as means and standard deviations as error bars. The same is shown in the graph on the right, but delivering an ERK1-specific shRNA (shERK1). Relevant statistical comparisons are shown within the graph. **c.** Phenotype effects of sustained MEK inhibition with PD0325901 on myeloid precursors committed to MDSC differentiation or to DC differentiation. Ly6C+ cells were gated and the Ly6G-CD11c expression profiles are shown in flow cytometry density plots. Percentages of CD11c+ myeloid cells after 7 days of culture are highlighted within the graphs. ***, very highly significant differences.

To assess the contribution of MDSC-associated kinases to myeloid differentiation, a collection of kinase inhibitors were added to myeloid precursors committed towards DC or B16-MDSC differentiation. Inhibitors were added at concentrations reported to interfere with cancer cell growth. High resolution impedance-based real-time cell monitoring (RTCA) was used to continuously monitor myeloid differentiation, viability and to calculate IC50s (Fig. 8b and Table 1) [15]. Overall, all tested inhibitors affected equally to myeloid precursors differentiating towards DCs and MDSCs (Table 1). Treatments with the specific AKT inhibitor X or the MEK inhibitor PD0325901 were an exception. AKT inhibitor X was highly toxic to precursors differentiating towards MDSCs, while differentiating DCs remained unaltered (Fig. 8b and Table 1). Treatment with the MEK inhibitor PD0325901 selectively inhibited MDSC proliferation. Overall, comparing the IC50s for differentiating DCs and MDSCs, AKT and MEK-ERK pathways specifically contributed to MDSC differentiation (Fig. 8c). Moreover, myeloid precursors committed to MDSC differentiation died within hours of adding the AKT inhibitor, strongly suggesting that AKT was involved in survival but not differentiation (Fig. 9a).

The results with kinase inhibitors were also confirmed with silencing shRNAs. Thus, myeloid precursors committed towards MDSC differentiation were expanded for three days from BM and transduced with a lentivector delivering immunoblot-validated shRNAs against AKT1 or ERK1 as described [8, 16] (Fig. 9b). Transduced myeloid precursors died 48 hours after delivery of the AKT1-specific shRNA. Likewise, ERK1 silencing significantly inhibited cell growth.

### Inhibition of the ERK pathway interferes with MDSC differentiation and accelerates DC maturation

Inhibition of the ERK pathway interfered with MDSC growth. As the MEK inhibitor PD0325901 is currently used for the treatment of several human cancers in clinical trials, its effects on differentiation of myeloid cell lineages was further tested. Thus, the three main myeloid cell populations differentiated from bone marrow precursors was quantified by flow cytometry after a week of PD0325901 treatment; Namely, CD11b^+^ monocytic myeloid cells (Ly6C^high^ Ly6G^neg^), granulocytic myeloid cells (Ly6C^+^ Ly6G^high^) and conventional DCs (Ly6C^+^ Ly6G^neg/low^ CD11c^+^) (Fig. 9c). Interestingly, PD0325901 treatment accelerated conventional CD11c^+^ DC differentiation. At the highest tested concentration, the MEK inhibitor was strongly cytotoxic to myeloid cells committed to MDSC differentiation, but not to those differentiating towards DCs which strongly up-regulated CD11c expression.

Overall, these results also confirmed that interference with the ERK pathway is inhibitory over MDSCs and promotes conventional DC differentiation.

## DISCUSSION

High-throughput analyses of biological systems provide a unified view of biological processes, and can uncover novel molecular targets. However, sorting out meaningful information from large datasets is challenging and relies on choosing the right controls. In addition, some biological systems such as MDSCs are difficult to work with. Here we characterized the neoplastic B16 melanoma MDSC proteome by differentiating MDSCs modeling tumor-infiltrating subsets, and quantitatively comparing it with the proteomes of myeloid DCs and MDSCs modeling non-cancerous MDSCs. Myeloid DCs and NC-MDSCs provide the appropriate controls to discriminate pathways regulated by cell lineage or the tumor environment. We performed in-depth quantitative proteomics to construct highly detailed MDSC interactome maps. Regulatory networks were for the first time unambiguously ascribed to cell lineage or to a neoplastic environment.

Cell lineage differences were highlighted by comparing B16 melanoma-MDSCs with myeloid DCs. Mitochondrial dysfunction was a key characteristic of MDSCs, reflecting a shut-down of oxidative phosphorylation. MDSCs modeling non-cancerous subsets provided a convenient standard to discriminate cancer-specific pathways. Of these, the pentose phosphate pathway was one of the most prominent, probably used to produce NADPH for biosynthesis in the absence of oxidative phosphorylation. Decreased expression of mitochondrial NADPH dehydrogenase, up-regulation of free radical scavenging proteins, and cellular stress were hallmarks of neoplastic MDSCs compared to NC-MDSCs. As we found that NC-MDSCs differed from neoplastic MDSCs in cell stress pathways and inflammatory pathways, it is likely that NC-MDSCs are different to other subsets differentiated in non-neoplastic conditions such as cell stress and infection. Overall, published data agreed with our proteome maps [17–24].

Importantly, neoplastic MDSCs presented a specific kinase signature which controlled MDSC-related interactomes and clearly separated them from the myeloid DC lineage. While SRC, FYN, HCK, PI3K and AKT kinases differentiated MDSCs from DCs, ERK and PKC discriminated neoplastic MDSCs from non-cancerous subsets. The proteomic and systems biology data was confirmed by immunoblot and flow cytometry. Ingenuity analyses also predicted the PKC isoforms as a differential feature of neoplastic MDSCs. DCs and NC-MDSCs presented lower but detectable levels of phospho-PKC. As we used a pan-phospho-PCK antibody, we cannot rule out that some specific PKC isoforms discriminate neoplastic MDSCs from non-neoplastic counterparts. In fact, this is the case of 4T1 breast cancer MDSCs, for which there is evidence that isoforms beta and theta are specifically activated [22].

AKT was required for the survival of myeloid precursors differentiating into MDSCs, but was dispensable in precursors committed to DC differentiation. This is in agreement with the requirement of AKT for *in vivo* MDSC expansion [25], and with its anti-apoptotic role in hematopoietic cells [26]. Additionally, the MEK1 inhibitor PD032591 selectively affected differentiating MDSCs, while DC maturation was enhanced according to CD11c up-regulation. The ERK pathway is known to keep DCs immature and favor tumor progression [27–29]. Here we also demonstrated that ERK contributes to MDSC differentiation. Our results show that inhibition of ERK and AKT pathways could enhance anti-tumor immune responses by depleting MDSCs and activating DCs. Nevertheless, the other differentially expressed kinases may still participate in MDSC functions apart from differentiation and survival, which could be susceptible of therapeutic intervention.

## MATERIALS AND METHODS

### Cells and mice

293T, B16F0 cells and BM-DCs were grown as described [8, 27]. Approval for animal studies was obtained from the Animal Ethics Committee of the University of Navarra, and from the Government of Navarra. Non-cancerous MDSCs (NC-MDSCs, 293T-MDSCs) and B16-MDSCs were obtained from C57BL/6 murine BM cells as described [8].

### Drug treatments of myeloid cell cultures and impedance-based real-time living cell monitoring (RTCA)

Myeloid hematopoietic precursors were expanded from BM cells using granulocyte-monocyte-colony stimulating factor (GM-CSF), stem cell factor (SCF) and leukaemia inhibitory factor (LIF) for 2 to 3 days, following published conditions [8, 30]. Then, myeloid precursors were seeded on two L8 cell culture chambers for the xCELLingence RTCA monitoring system (ACEA biosciences), at a density of 200000 cells per well. DC or B16-MDSC differentiation medium was added to myeloid precursors, and treatments were carried out simultaneously in duplicates. After 30 min, the indicated chemical inhibitors were added at concentrations reported to be cytotoxic to cancer cells. Control wells were treated with carrier solution (either water or DMSO). The following inhibitors were used: AKT inhibitor X (Calbiochem), tyrosine kinase inhibitor TX-1123 (Calbiochem), MEK inhibitor PD0325901 (SIGMA), ERK inhibitors SCH772984 and VTX-11e [31], broad PKC inhibitor GÖ 6983 (Santa Cruz Biotechnology), PKC inhibitor NPC-15437 dihydrochloride (Santa Cruz Biotechnology), selective LCK and FYN inhibitor PP2 (Santa Cruz Biotechnology), and the SRC and FYN inhibitor Saracatinib (MedChem Express). IC50s for each inhibitor were calculated using the RTCA data and analysis software, using duplicates for each drug treatment.

### Lentivector production and cell transduction

Lentivectors were produced and titrated by flow cytometry or Q-PCR as described [32]. The pHIV-SIREN system developed by our group [16] was used as a backbone to clone the following validated shRNAs against ERK1 (GCATGCTTAATTCCAAGGGCTATTCAAGAGATAG CCCTTGGAATTAAGCATGTTTTTTACGCGT) and AKT1 (GTCTGAGACTGACACCAGGTATTTCAAGAG AATACCTGGTGTCAGTCTCAGATTTTTTACGCGT). A control shRNA-encoding lentivector targeting the human PD1 transcript (SIREN-shPD1a) was used [33]. The same shRNAs were cloned into the pSIRACT-GFP shRNA-cloning lentivectors, which were derived from pHIV-SIREN constructs by replacing the PGK promoter by the Actinin 4 promoter. The lentivector backbone was changed because PGK was strongly down-modulated in MDSCs, while actinin4 was strongly expressed.

### Immunoblot

Immunoblots were performed as described [27]. Anti-GADPH was purchased from Calbiochem. Rabbit anti-human HCK was purchased from Millipore. From BD bioscience, mouse anti-pan ERK, mouse anti-AKT and mouse anti-AKT pT308. From Cell Signaling, rabbit anti-mouse T202/Y204 p-P44/42 MAPK, phospho-pan-PKC rabbit mAb. Peroxidase-conjugated anti-mouse and anti-rabbit antibodies were purchased from DAKO. Membranes were stripped and re-probed with antibodies for total and phosphorylated proteins, when required.

### Cell staining and flow cytometry

Surface and intracellular staining were performed as described previously [27] using the indicated antibodies. From BioLegend: Alexa fluor 488-Ly6C, PE-Cy7-Ly6G, PE-Cy7-streptavidin, APC-streptatividin; From BD Pharmigen: APC-CD11b, PE-Cy7 anti-mouse CD11c, Rat anti-mouse CD16/CD32, PE-conjugated anti-Gr-1, Alexa 647-conjugated anti- PY418 SRC, PE-conjugated ant-AKT1, from Invitrogen: APC-CD11c, PE-streptavidin, FITC-streptavidin; from AbDSerotec: PE-CD62L; From Santa Cruz Biotechnology: PE-conjugated anti-Fyn. From Cell Signaling, Alexa 647-conjugated anti-SRC rabbit antibody (clone 36D10) and PE-conjugated anti-phospho AKT rabbit antibody (Ser473, clone D9E).

### Mass spectrometry-based quantitative proteomics and bioinformatic analyses

A global experiment was carried out with three biological replicates in each experimental condition using B16-MDSC, NC-MDSC and DC cell pellets. The specific procedures for sample preparation, proteomic analyses, iTRAQ-based proteomic workflows and mass spectrometry using triple-TOF 5600 system (AB Sciex) have been published [8]. The mass spectrometry proteomics data were deposited to the ProteomeXchange Consortium (http://proteomecentral.proteomexchange.org) via the PRIDE partner repository with the data set identifiers PXD001103 and PXD001106.

Analyses of raw data (.wiff, AB Sciex) were performed with *MaxQuant* software [34]. For peak list generation, default AB Sciex Q-TOF instrument parameters were used except the main search peptide tolerance, which was set to 0.01 Da, and MS/MS match tolerance, which was increased up to 50 ppm. Minimum peptide length was set to 6. Two databases were used. A contaminant database (.fasta) was firstly used for filtering out contaminants. Peak lists were searched against UniProt murine database, and Andromeda was used as a search engine [35]. Methionine oxidation was set as variable modification, and the carbamidomethylation of cysteine residues was set as fixed modification. Maximum false discovery rates (FDR) were set to 0.01 at protein and peptide levels. Analyses were limited to peptides of six or more amino acids in length, and considering a maximum of two missed cleavages. Relative protein abundance output data files were managed using R scripts for subsequent statistical analyses and representation. Proteins identified by site (identification based only on a modification), reverse proteins (identified by decoy database) and potential contaminants were filtered out. Only proteins with more than one identified peptide were used for quantification. For possible quantification data rescue, up to one missing value for each group was rescued replacing it by the mean of the rest in-group samples. Data was normalized and transformed for later comparison using quantiles normalization and log2 transform respectively. The Limma Bioconductor software package in R was used for ANOVA analyses. Significant and differential data were selected by a *p*-value lower than 0.01, fold changes of <0.77 (down-regulation) and >1.3 (up-regulation) in linear scale. These parameters were used for differential expression threshold with volcano and profile plots.

The proteomic information was analyzed using bioinformatic tools. Studies with the Kyoto Encyclopedia of Genes and Genomes (KEGGS) Pathway mapping tool were performed as described (http://www.genome.jp/kegg/tool/map_pathway1.html). The identification of specifically up- or dysregulated regulatory/metabolic networks in MDSCs was analyzed with the open access STRING (Search Tool for the Retrieval of Interacting Genes) analysis tool (v.9.1) [14] and with the Ingenuity Pathway Analysis Tool (Qiagen).

### Statistical analyses

GraphPad Prism and SPSS software packages were used for plotting data and statistical analyses. No data was considered an outlier. Real time cell monitoring data (RTCA, ACEA biosystems) was analyzed by exporting the Cell Index data as a function of time. It was confirmed that Cell Index in a growing population of cells was highly homogeneous and normally distributed at any given time-point. Therefore, the data was analyzed by one-way ANOVA and Tukey’s pair-wise comparisons. IC50s were estimated for each treatment (using three published active concentrations per compound) in duplicates by RTCA, and means with standard deviations were obtained. IC50s were also highly homogeneous and normally distributed. The relative IC50 ratios for DCs vs MDSCs were also calculated, and compared by one-way ANOVA and Tukey’s pair wise comparisons. Cell viability was quantified by trypan blue staining and data analyzed by one-way ANOVA and Tukey’s pair wise comparisons. Triplicates per treatment were used for the analyses. Growth of myeloid cells transduced with lentivectors encoding shRNAs was compared by a two-way ANOVA with “time of growth” as a random factor with data from four independent transductions, as described previously [8].

## SUPPLEMENTARY FIGURES AND TABLE


