# Supplementary material for: A core of kinase-regulated interactomes defines the neoplastic MDSC lineage
Source: Oncotarget. 2015 Jul 23;6(29):27160–75. doi: 10.18632/oncotarget.4746 (PMC4694980; doi:10.18632/oncotarget.4746)
Supplement: Supplementary file 1 [file oncotarget-06-27160-s001.pdf]

## SUPPLEMENTARY FIGURES AND TABLE

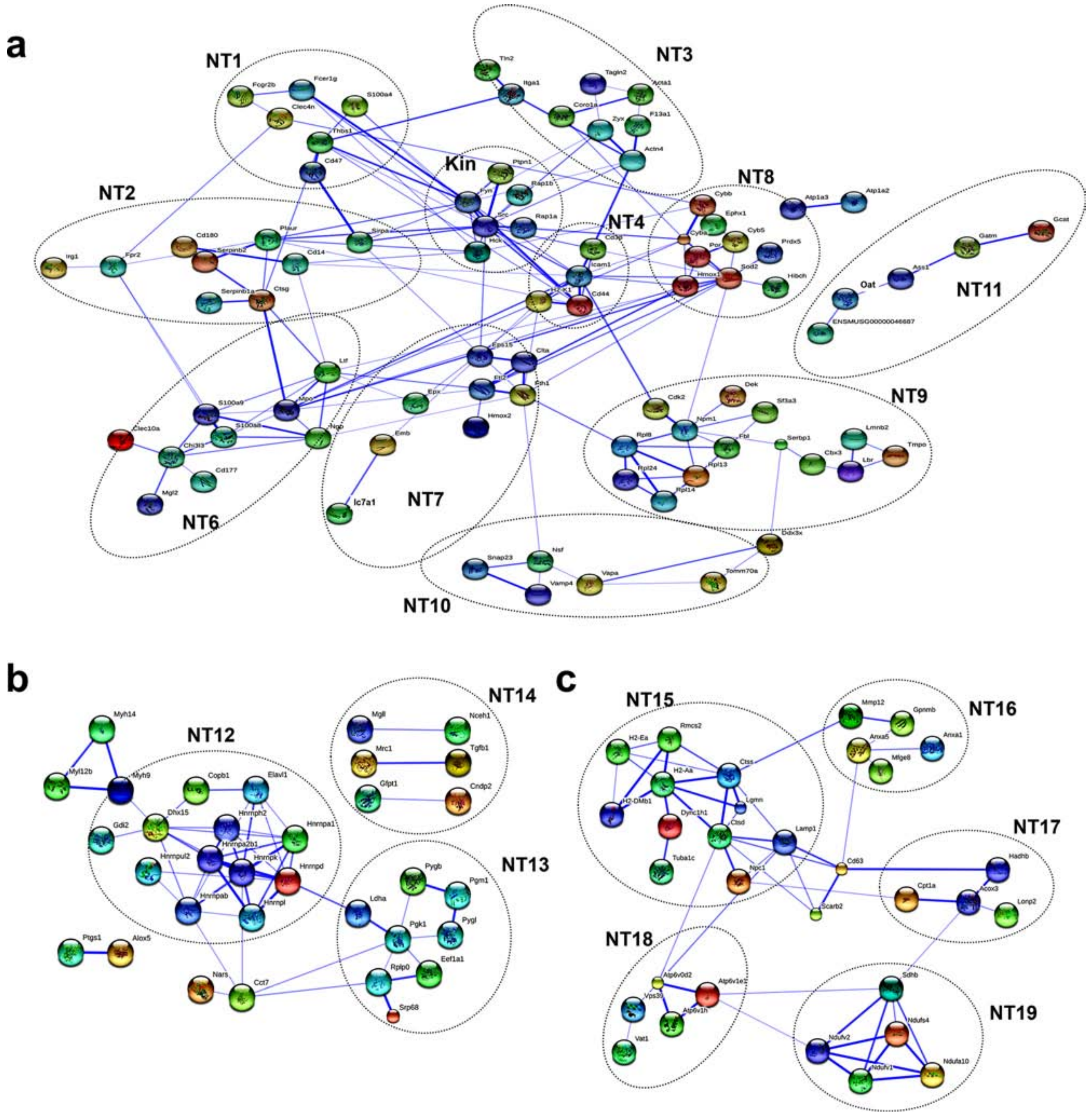


**Supplementary Figure S1: STRING functional interactomes with lineage-specific differentially expressed proteins (B16-MDSC vs DCs). a.** Interactome using differentially up-regulated proteins as inputs. Medium (thin lines, score of 0.400) and high (thick lines, score of 0.700) confidence relationships between protein nodes are indicated in the graph. Internodal relationships were independently confirmed. Proteins were encircled and grouped as interconnected networks (NTs) to facilitate interpretation. A central network of kinases (Kin) is shown in the middle, which links the other networks. Networks NT1, NT2, NT3 and NT4 grouped membrane receptors together with associated signal transduction proteins. Networks NT6 and NT7 included S100A protein family members, c-type lectins, membrane receptors involved in phagocyte migration to sites of inflammation and phagocyte-associated enzymes. NT8 included redox proteins associated to reactive oxygen species (ROS) and protection against oxidative damage and xenobiotics. NT9 comprised ribosomal proteins and regulators of nuclear processes and cell division. NT10 included proteins involved in intracellular vesicle trafficking, while NT11 consisted in a network of aminoacid metabolic pathways. **b.** Interactome map grouping differentially down-modulated proteins involved in splicesosome formation (NT12) and carbon metabolism (NT13). **c.** As in (b) with down-modulated proteins involved in MHC II antigen presentation (NT15), lysosomal functions (NT18) and mitochondrial complex I (NT19).

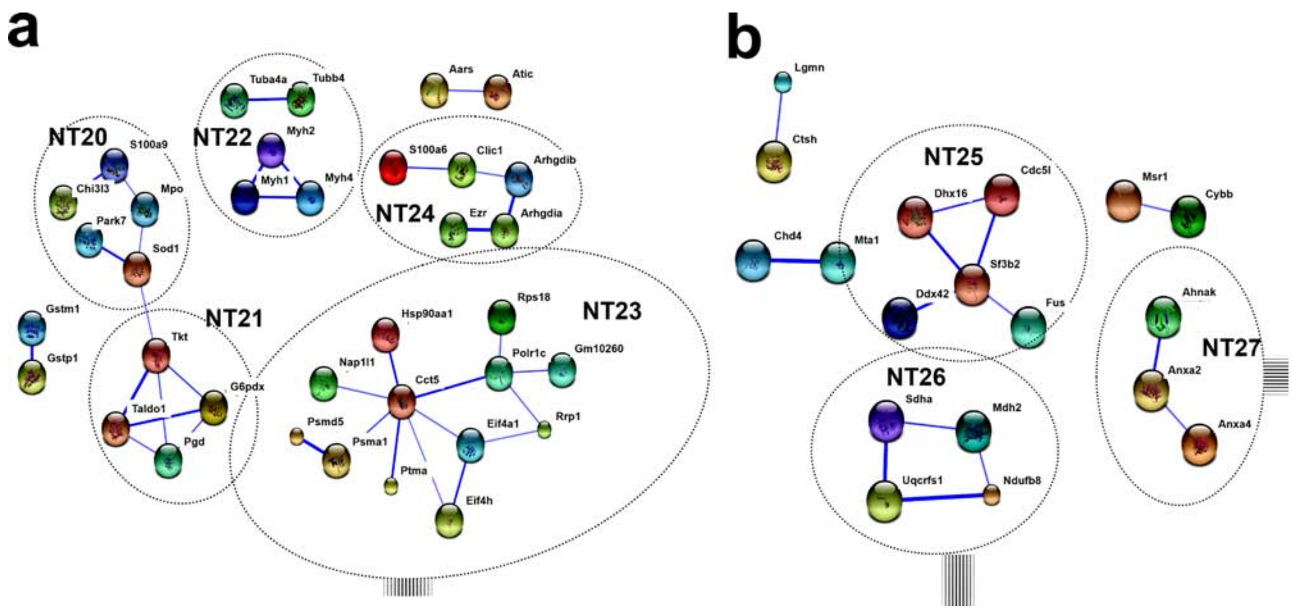


**Supplementary Figure S2: STRING Functional interactomes with tumor-regulated (B16-MDSCs vs NC-MDSCs) differentially expressed proteins. a.** STRING interactomes with up-regulated proteins. Medium (thin lines, score of 0.400) and high (thick lines, score of 0.700) confidence relationships between protein nodes are indicated in the graph. Internodal relationships were independently confirmed. Proteins were encircled and grouped as interconnected networks (NTs) to facilitate interpretation. NT20 grouped proteins involved in inflammatory receptor signaling and ROS scavenging proteins (SOD2, PARK7, MPO). NT21 grouped the pentose phosphate pathway. NT22 contained tubulin and myosin, pointing to changes in the cell cytoskeleton. NT23 included proteins involved in ribosomal RNA transcription, protein translation, folding and proteosomal degradation. NT24 contained proteins involved in membrane signaling, membrane structure and cytoskeleton rearrangements. **b.** As in (a), NT25 contained proteins involved in gene expression and splicesosome organization. NT26 contained mitochondrial NADH dehydrogenase complex I and complex III proteins. NT27 contained cell growth-promoting and signal transduction proteins.

## Supplementary Table S1. *Differentially expressed proteins*

| B16-MDSC vs DC differentially expressed proteins | | | | |
|---|---|---|---|---|
| **Protein names** | **Gene names** | **Peptides** | **Fold change** | ***P* value** |
| Annexin A5 | Anxa5 | 24 | 0, 409148995 | 6, 08E-07 |
| Rho guanine nucleotide exchange factor 2 | Arhgef2 | 2 | 0, 414308614 | 6, 6E-05 |
| Lactadherin | Mfge8 | 11 | 0, 429506653 | 4, 11E-06 |
| Annexin A1 | Anxa1 | 25 | 0, 481617476 | 6, 04E-05 |
| Twinfilin-1 | Twf1 | 2 | 0, 489112815 | 0, 000865 |
| Tubulin alpha-1C chain | Tuba1c | 18 | 0, 49021701 | 0, 0008 |
| Class II histocompatibility antigen, M beta 1 chain | H2-DMb1;H2-DMb2 | 2 | 0, 491533209 | 8, 1E-05 |
| H-2 class II histocompatibility antigen, E-U alpha chain | H2-Ea | 5 | 0, 498582303 | 8, 08E-06 |
| H-2 class II histocompatibility antigen, A-D alpha chain | H2-Aa | 4 | 0, 522877994 | 6, 45E-06 |
| Myosin regulatory light chain 12B | Myl12b;Myl9 | 9 | 0, 536878005 | 0, 000273 |
| Glycogen phosphorylase, brain form | Pygb;Pygm | 3 | 0, 538066216 | 0, 001733 |
| Lon protease homolog 2, peroxisomal | Lonp2 | 2 | 0, 538215926 | 0, 000881 |
| Solute carrier family 35 member F6 | Slc35f6 | 2 | 0, 543109435 | 0, 003618 |
| H-2 class II histocompatibility antigen, E-D beta chain | | 5 | 0, 557078509 | 1, 3E-05 |
| Asparagine—tRNA ligase, cytoplasmic | Nars | 4 | 0, 565052924 | 0, 000702 |
| CD63 antigen | Cd63 | 2 | 0, 578425521 | 0, 003113 |
| Fatty acid-binding protein, epidermal | Fabp5 | 3 | 0, 600473325 | 0, 000207 |
| H-2 class II histocompatibility antigen, A-D beta chain | H2-Ab1 | 9 | 0, 609153728 | 7, 78E-05 |
| Myosin-9 | Myh9 | 111 | 0, 611970692 | 0, 000644 |
| V-type proton ATPase subunit d 2 | Atp6v0d2 | 7 | 0, 630823671 | 0, 000251 |
| Heterogeneous nuclear ribonucleoproteins A2/B1 | Hnrnpa2b1 | 14 | 0, 63694467 | 5, 2E-05 |
| Myosin-14 | Myh14 | 7 | 0, 642878205 | 0, 0003 |

(*Continued*)

| B16-MDSC vs DC differentially expressed proteins | | | | |
|---|---|---|---|---|
| **Protein names** | **Gene names** | **Peptides** | **Fold change** | ***P* value** |
| Uncharacterized protein | Gm8730;Rplp0 | 14 | 0, 648606361 | 0, 000193 |
| Neutral cholesterol ester hydrolase 1 | Nceh1 | 7 | 0, 651305733 | 4, 73E-05 |
| Heterogeneous nuclear ribonucleoprotein A/B | Hnrnpab | 7 | 0, 65477646 | 7, 23E-05 |
| Monoglyceride lipase | Mgll | 2 | 0, 657831546 | 0, 00342 |
| Transmembrane glycoprotein NMB | Gpnmb | 6 | 0, 66221021 | 9, 17E-05 |
| von Willebrand factor A domain-containing protein 5A | Vwa5a | 4 | 0, 663896759 | 0, 001158 |
| Monoacylglycerol lipase ABHD12 | Abhd12 | 6 | 0, 677723053 | 0, 003818 |
| Ras-related GTP-binding protein C | Rragd;Rragc | 2 | 0, 678167689 | 0, 004017 |
| Lysosome membrane protein 2 | Scarb2 | 4 | 0, 679787707 | 0, 000251 |
| Heterogeneous nuclear ribonucleoprotein U-like protein 2 | Hnrnpul2 | 7 | 0, 685215928 | 0, 001169 |
| Cathepsin D | Ctsd | 13 | 0, 691475625 | 0, 000874 |
| Putative sodium-coupled neutral amino acid transporter 10 | Slc38a10 | 3 | 0, 693741006 | 0, 004065 |
| Signal recognition particle subunit SRP68 | Srp68 | 2 | 0, 6969969 | 0, 00223 |
| Alpha-2-macroglobulin receptor-associated protein | Lrpap1 | 20 | 0, 698359835 | 0, 000132 |
| Elongation factor 1-alpha 1 | Eef1a1;Eef1a2 | 18 | 0, 700559018 | 0, 000256 |
| Cathepsin S | Ctss | 6 | 0, 701751582 | 0, 001797 |
| Estradiol 17-beta-dehydrogenase 11 | Hsd17b11 | 4 | 0, 703096327 | 0, 000583 |
| Succinate dehydrogenase [ubiquinone] iron-sulfur subunit, mitochondrial | Sdhb | 9 | 0, 706493357 | 0, 000214 |
| Transforming growth factor beta-1 | Tgfb1 | 2 | 0, 707417988 | 0, 002654 |
| Tropomyosin alpha-3 chain | Tpm3;Tpm3-rs7 | 21 | 0, 7087286 | 0, 006006 |

(*Continued*)

| B16-MDSC vs DC differentially expressed proteins | | | | |
|---|---|---|---|---|
| **Protein names** | **Gene names** | **Peptides** | **Fold change** | ***P* value** |
| WASH complex subunit strumpellin | Kiaa0196 | 2 | 0, 710467852 | 0, 006814 |
| Lysosome-associated membrane glycoprotein 1 | Lamp1 | 4 | 0, 718359696 | 0, 003244 |
| ELAV-like protein 1 | Elavl1 | 5 | 0, 720005557 | 0, 000596 |
| LEM domain-containing protein 2 | Lemd2 | 4 | 0, 723410824 | 0, 002332 |
| Aminopeptidase N | Anpep | 26 | 0, 724568989 | 0, 000451 |
| Heterogeneous nuclear ribonucleoprotein A1 | Hnrnpa1 | 8 | 0, 728604525 | 0, 000505 |
| NADH dehydrogenase [ubiquinone] 1 alpha subcomplex subunit 10, mitochondrial | Ndufa10 | 6 | 0, 730876406 | 0, 003613 |
| Heterogeneous nuclear ribonucleoprotein H2 | Hnrnph2 | 6 | 0, 731481958 | 0, 000661 |
| Legumain | Lgmn | 3 | 0, 733714602 | 0, 00072 |
| ER membrane protein complex subunit 1 | Emc1 | 6 | 0, 736539664 | 0, 006507 |
| Serpin B6 | Serpinb6a;Serpinb6 | 8 | 0, 739418148 | 0, 002021 |
| Nicastrin | Ncstn | 5 | 0, 739929041 | 0, 001134 |
| Cytoplasmic dynein 1 heavy chain 1 | Dync1 h1 | 36 | 0, 746509337 | 0, 000733 |
| Macrophage metalloelastase | Mmp12 | 11 | 0, 747676988 | 0, 003364 |
| L-amino-acid oxidase | Il4i1 | 4 | 0, 748410986 | 0, 003715 |
| DnaJ homolog subfamily C member 11 | Dnajc11 | 3 | 0, 74862864 | 0, 001608 |
| Glutamine—fructose-6-phosphate aminotransferase [isomerizing] 1 | Gfpt1 | 4 | 0, 749338067 | 0, 0075 |
| Lymphocyte antigen 75 | Ly75 | 4 | 0, 750276114 | 0, 007796 |
| Macrophage mannose receptor 1 | Mrc1 | 18 | 0, 750401561 | 0, 003811 |
| Golgi membrane protein 1 | Golm1 | 2 | 0, 751319227 | 0, 001423 |
| Dipeptidyl peptidase 2 | Dpp7 | 2 | 0, 751371795 | 0, 002028 |
| Vam6/Vps39-like protein | Vps39 | 2 | 0, 753460592 | 0, 001331 |

(*Continued*)

| B16-MDSC vs DC differentially expressed proteins | | | | |
|---|---|---|---|---|
| **Protein names** | **Gene names** | **Peptides** | **Fold change** | ***P* value** |
| Amine oxidase [flavin-containing] A | Maoa | 3 | 0, 753928115 | 0, 007226 |
| Creatine kinase B-type | Ckb | 8 | 0, 754517987 | 0, 001915 |
| Nicotinamide phosphoribosyltransferase | Nampt | 2 | 0, 755875358 | 0, 00465 |
| Inosine triphosphate pyrophosphatase | Itpa | 2 | 0, 760176881 | 0, 008895 |
| Heterogeneous nuclear ribonucleoprotein L | Hnrnpl | 7 | 0, 76047527 | 0, 001026 |
| Ribonuclease inhibitor | Rnh1 | 7 | 0, 760502021 | 0, 000888 |
| B-cell receptor-associated protein 31 | Bcap31 | 11 | 0, 761263852 | 0, 00613 |
| NADH dehydrogenase [ubiquinone] iron-sulfur protein 4, mitochondrial | Ndufs4 | 5 | 0, 762621458 | 0, 005491 |
| V-type proton ATPase subunit E 1 | Atp6v1e1 | 16 | 0, 763478047 | 0, 004022 |
| Heterogeneous nuclear ribonucleoprotein K | Hnrnpk;Gm7964 | 12 | 0, 763856162 | 0, 005636 |
| L-lactate dehydrogenase | Ldha | 13 | 0, 764291243 | 0, 000965 |
| Putative pre-mRNA-splicing factor ATP-dependent RNA helicase DHX15 | Dhx15 | 7 | 0, 765273038 | 0, 001402 |
| Phosphoglycerate kinase 1 | Pgk1 | 18 | 0, 766086802 | 0, 007756 |
| Glycogen phosphorylase, liver form | Pygl | 16 | 0, 766633266 | 0, 0034 |
| NADH dehydrogenase [ubiquinone] flavoprotein 2, mitochondrial | Ndufv2 | 7 | 0, 76712425 | 0, 004833 |
| NADH dehydrogenase [ubiquinone] flavoprotein 1, mitochondrial | Ndufv1 | 10 | 0, 769871468 | 0, 004625 |
| Mimitin, mitochondrial | Ndufaf2 | 2 | 1, 30381531 | 0, 002611 |
| Mitochondrial import receptor subunit TOM70 | Tomm70a | 7 | 1, 304563258 | 0, 001894 |
| Ras-related protein Rap-1b | Rap1b;Rap1a | 6 | 1, 307217616 | 0, 003029 |
| Tyrosine-protein phosphatase non-receptor type 1 | Ptpn1 | 12 | 1, 310480124 | 0, 001443 |

(*Continued*)

| B16-MDSC vs DC differentially expressed proteins | | | | |
|---|---|---|---|---|
| **Protein names** | **Gene names** | **Peptides** | **Fold change** | ***P* value** |
| Lactotransferrin | Ltf | 6 | 1, 311085 | 0, 009649 |
| Vacuolar protein sorting-associated protein 33A | Vps33a | 2 | 1, 317391275 | 0, 000905 |
| Mitochondrial import inner membrane translocase subunit Tim8 A | Timm8a1;Timm8a2 | 2 | 1, 322616616 | 0, 003141 |
| Argininosuccinate synthase | Ass1;Gm5424 | 9 | 1, 323069518 | 0, 004604 |
| Ferritin | Ftl1;Ftl2 | 10 | 1, 323319619 | 0, 001567 |
| Protein S100-A4 | S100a4 | 3 | 1, 326852892 | 0, 002104 |
| Nucleophosmin | Npm1;Gm5611 | 14 | 1, 328008535 | 0, 000918 |
| Integrin alpha-1 | Itga1 | 5 | 1, 33234292 | 0, 008921 |
| Epidermal growth factor receptor substrate 15 | Eps15 | 4 | 1, 335132857 | 0, 007084 |
| Adenylate kinase 2, mitochondrial | Ak2 | 9 | 1, 339117363 | 0, 000355 |
| Lamin-B receptor | Lbr | 10 | 1, 340226149 | 0, 002185 |
| Heme oxygenase 1 | Hmox1 | 7 | 1, 341204992 | 0, 00124 |
| Platelet receptor Gi24 | 4632428N05Rik | 4 | 1, 350347861 | 0, 002464 |
| Transgelin-2 | Tagln2 | 12 | 1, 351776016 | 0, 001362 |
| Low affinity immunoglobulin gamma Fc region receptor II | Fcgr2 | 6 | 1, 366625261 | 0, 002449 |
| Leukocyte elastase inhibitor A | Serpinb1a | 6 | 1, 37092156 | 0, 001528 |
| Perilipin-3 | Plin3 | 3 | 1, 373402061 | 0, 002905 |
| Superoxide dismutase [Mn], mitochondrial | Sod2 | 8 | 1, 373760594 | 0, 001123 |
| High affinity immunoglobulin epsilon receptor subunit gamma | Fcer1g | 4 | 1, 374118744 | 0, 00872 |
| Embigin | Emb | 4 | 1, 377538576 | 0, 001189 |
| Formyl peptide receptor 2 | Fpr2 | 3 | 1, 379093299 | 0, 000644 |
| Cytochrome b5 | Cyb5a | 6 | 1, 380498718 | 0, 001192 |
| Chromobox protein homolog 3 | Cbx3 | 6 | 1, 380952591 | 0, 001884 |
| Acid sphingomyelinase-like phosphodiesterase 3b | Smpdl3b | 4 | 1, 384287037 | 0, 002606 |

(*Continued*)

| B16-MDSC vs DC differentially expressed proteins | | | | |
|---|---|---|---|---|
| **Protein names** | **Gene names** | **Peptides** | **Fold change** | ***P* value** |
| Protein-arginine deiminase type-4 | Padi4 | 9 | 1, 40155117 | 0, 000424 |
| Protein LYRIC | Mtdh | 7 | 1, 406115785 | 0, 002098 |
| Bcl-2-like protein 13 | Bcl2l13 | 3 | 1, 408599571 | 0, 001685 |
| H-2 class I histocompatibility antigen, K-B alpha chain | H2-K1 | 8 | 1, 41010511 | 0, 001533 |
| Napsin-A | Napsa | 2 | 1, 411600269 | 0, 005092 |
| Coronin-1A | Coro1a | 12 | 1, 414013879 | 0, 00018 |
| 2-amino-3-ketobutyrate coenzyme A ligase, mitochondrial | Gcat | 2 | 1, 421520698 | 0, 006462 |
| Cyclin-dependent kinase 2 | Cdk2 | 2 | 1, 426124115 | 0, 009262 |
| Plexin domain-containing protein 2 | Plxdc2 | 5 | 1, 42751035 | 0, 00033 |
| Rho GTPase-activating protein 1 | Arhgap1 | 5 | 1, 428460911 | 0, 000189 |
| Histone H1.0 | H1f0 | 3 | 1, 429352055 | 0, 00982 |
| Peroxiredoxin-5, mitochondrial | Prdx5 | 10 | 1, 43101649 | 0, 000163 |
| Cytochrome b-245 light chain | Cyba | 5 | 1, 432458891 | 0, 000559 |
| CD44 antigen | Cd44 | 3 | 1, 443457084 | 0, 000215 |
| Zyxin | Zyx | 4 | 1, 444345746 | 0, 008419 |
| Cytochrome b-245 heavy chain | Cybb | 10 | 1, 455588804 | 0, 000222 |
| Chitinase-like protein 3 | Chil3 | 18 | 1, 46074136 | 0, 000102 |
| Tyrosine-protein kinase HCK | Hck | 5 | 1, 464103351 | 0, 000238 |
| Leukocyte surface antigen CD47 | Cd47 | 2 | 1, 465022748 | 0, 003868 |
| C-type lectin domain family 6 member A | Clec4n;Clec6a | 2 | 1, 467063188 | 0, 00017 |
| CD177 antigen | Cd177 | 5 | 1, 473425935 | 0, 002559 |
| Lamina-associated polypeptide 2, isoforms beta/delta/epsilon/gamma | Tmpo | 11 | 1, 476178931 | 0, 00123 |
| Glycerol kinase | Gyk | 2 | 1, 481175737 | 0, 000568 |
| Alpha-actinin-4 | Actn4 | 36 | 1, 485459058 | 7, 33E-05 |

(*Continued*)

| B16-MDSC vs DC differentially expressed proteins | | | | |
|---|---|---|---|---|
| **Protein names** | **Gene names** | **Peptides** | **Fold change** | ***P* value** |
| ATP-dependent (S)-NAD(P)H-hydrate dehydratase | Carkd | 2 | 1, 487792072 | 0, 004226 |
| Actin, alpha skeletal muscle | Acta1 | 17 | 1, 490499494 | 0, 001138 |
| Plasminogen activator inhibitor 1 RNA-binding protein | Serbp1 | 7 | 1, 515466638 | 0, 000249 |
| Tyrosine-protein kinase Fyn | Fyn;Yes1 | 3 | 1, 524885079 | 0, 003341 |
| Sodium/potassium-transporting ATPase subunit alpha-3 | Atp1a3;Atp1a2 | 13 | 1, 525120684 | 0, 002015 |
| 60S ribosomal protein L8 | Rpl8 | 5 | 1, 526316859 | 0, 00643 |
| Urokinase plasminogen activator surface receptor | Plaur | 2 | 1, 529089244 | 0, 000475 |
| Vesicle-associated membrane protein 4 | Vamp4 | 3 | 1, 538993145 | 0, 001874 |
| ADP-ribosyl cyclase/cyclic ADP-ribose hydrolase 1 | Cd38 | 3 | 1, 539999685 | 0, 000433 |
| 60S ribosomal protein L24 | Rpl24;Gm17430 | 4 | 1, 542903394 | 0, 009811 |
| Plasminogen activator inhibitor 2, macrophage | Serpinb2 | 3 | 1, 562731137 | 0, 000522 |
| 60S ribosomal protein L13 | Rpl13 | 3 | 1, 568815619 | 0, 000839 |
| Coagulation factor XIII A chain | F13a1 | 5 | 1, 575022778 | 0, 002436 |
| Protein S100-A9 | S100a9 | 5 | 1, 585262138 | 0, 000482 |
| C-type lectin domain family 10 member A | Clec10a | 9 | 1, 608439704 | 0, 000107 |
| 3-hydroxyisobutyryl-CoA hydrolase, mitochondrial | Hibch | 2 | 1, 633954389 | 0, 002052 |
| Neuronal proto-oncogene tyrosine-protein kinase Src | Src | 3 | 1, 641084449 | 0, 000776 |
| Macrophage galactose N-acetyl-galactosamine specific lectin 2 | Mgl2 | 13 | 1, 648910806 | 3, 73E-05 |
| Monocyte differentiation antigen CD14 | Cd14 | 3 | 1, 649302138 | 0, 000391 |

(*Continued*)

| B16-MDSC vs DC differentially expressed proteins | | | | |
|---|---|---|---|---|
| **Protein names** | **Gene names** | **Peptides** | **Fold change** | ***P* value** |
| Signal-regulatory protein alpha | Sirpa | 5 | 1, 667987603 | 0, 007842 |
| High affinity cationic amino acid transporter 1 | Slc7a1 | 2 | 1, 683193993 | 0, 002121 |
| Cathepsin G | Ctsg | 2 | 1, 684502903 | 0, 000662 |
| Zinc transporter ZIP4 | Slc39a4 | 2 | 1, 701167303 | 0, 000458 |
| Ferritin heavy chain | Fth1 | 3 | 1, 70872916 | 4, 24E-05 |
| Phostensin | Ppp1r18 | 4 | 1, 734747128 | 0, 001686 |
| Myeloperoxidase | Mpo | 14 | 1, 739785165 | 1, 48E-05 |
| Citrate lyase subunit beta-like protein, mitochondrial | Clybl | 2 | 1, 812987713 | 0, 00626 |
| Brain acid soluble protein 1 | Basp1 | 8 | 1, 855107248 | 8, 3E-05 |
| Carbonic anhydrase 4 | Ca4;Car4 | 10 | 1, 910244551 | 4, 79E-06 |
| Lymphocyte-specific protein 1 | Lsp1 | 12 | 1, 914761621 | 2, 34E-06 |
| Protein DEK | Dek | 3 | 1, 924824794 | 6, 07E-05 |
| Talin-2 | Tln2 | 7 | 1, 958388414 | 0, 002108 |
| Protein Ahnak | Ahnak | 95 | 2, 08080018 | 2, 1E-06 |
| 60S ribosomal protein L14 | Rpl14 | 3 | 2, 097473177 | 0, 00187 |
| Protein S100-A8 | S100a8 | 6 | 2, 162403323 | 6, 12E-05 |
| Eosinophil peroxidase | Epx | 16 | 2, 166652695 | 3, 09E-05 |
| Myeloid bactenecin (F1) | Ngp | 3 | 2, 254673881 | 4, 64E-06 |
| Interferon-induced transmembrane protein 3 | Ifitm3 | 3 | 2, 270237035 | 2, 09E-06 |

| NC-MDSC vs B16-MDSC differentially expressed proteins | | | | |
|---|---|---|---|---|
| **Protein names** | **Gene names** | **Peptides** | **Fold change** | ***P* value** |
| Myosin-4 | Myh2;Myh4;Myh1 | 3 | 0, 272566219 | 0, 00017 |
| Lymphocyte-specific protein 1 | Lsp1 | 6 | 0, 421060939 | 0, 000113 |
| MCG130173 | Stfa2l1 | 2 | 0, 513643456 | 4, 06E-05 |
| 2010005H15Rik protein | 2010005H15Rik | 3 | 0, 525149229 | 5, 18E-05 |
| Chitinase-like protein 3 | Chil3 | 12 | 0, 550932031 | 0, 000314 |
| D-3-phosphoglycerate dehydrogenase | Phgdh | 5 | 0, 564754318 | 8, 73E-05 |
| MCG130175, isoform CRA_b | BC100530 | 5 | 0, 579007568 | 8, 97E-05 |

(*Continued*)

| NC-MDSC vs B16-MDSC differentially expressed proteins | | | | |
|---|---|---|---|---|
| **Protein names** | **Gene names** | **Peptides** | **Fold change** | ***P* value** |
| MCG130182, isoform CRA_a | Gm5483 | 3 | 0, 589206137 | 0, 000126 |
| 26S proteasome non-ATPase regulatory subunit 5 | Psmd5 | 2 | 0, 598593317 | 0, 000267 |
| Eukaryotic translation initiation factor 4H | Eif4 h | 4 | 0, 611736747 | 0, 001747 |
| CapZ-interacting protein | Rcsd1 | 3 | 0, 612295734 | 0, 000745 |
| Protein S100-A6 | S100a6 | 2 | 0, 617006908 | 0, 008245 |
| Rho GDP-dissociation inhibitor 2 | Arhgdib | 7 | 0, 618770272 | 0, 000466 |
| Tubulin alpha-4A chain | Tuba4a | 10 | 0, 626186143 | 0, 003667 |
| Superoxide dismutase [Cu-Zn] | Sod1 | 3 | 0, 631095674 | 0, 000772 |
| Nucleosome assembly protein 1-like 1 | Nap1l1 | 5 | 0, 640182294 | 0, 004251 |
| Protein S100-A9 | S100a9 | 5 | 0, 648160088 | 0, 001437 |
| Prothymosin alpha | Ptma | 3 | 0, 651999552 | 0, 00052 |
| Myosin light polypeptide 6 | Myl6 | 8 | 0, 656424969 | 0, 005285 |
| Astrocytic phosphoprotein PEA-15 | Pea15 | 2 | 0, 666877728 | 0, 003191 |
| DNA-directed RNA polymerases I and III subunit RPAC1 | Polr1c | 2 | 0, 67045724 | 0, 006728 |
| Glucose-6-phosphate 1-dehydrogenase X | G6pdx | 13 | 0, 670989902 | 0, 001161 |
| Spermine synthase | Sms | 2 | 0, 673833379 | 0, 005298 |
| Leukotriene A-4 hydrolase | Lta4 h | 13 | 0, 686384982 | 0, 001508 |
| Myeloperoxidase | Mpo | 7 | 0, 688935214 | 0, 005634 |
| Alpha-N-acetylglucosaminidase | Naglu | 3 | 0, 6900478 | 0, 009688 |
| Eukaryotic initiation factor 4A-I | Eif4a1 | 13 | 0, 694111659 | 0, 001646 |
| Tubulin beta-4B chain | Tubb4b;Tubb4a | 14 | 0, 696428165 | 0, 005599 |
| Transaldolase | Taldo1 | 10 | 0, 703041508 | 0, 001204 |
| Rho GDP-dissociation inhibitor 1 | Arhgdia | 5 | 0, 708523919 | 0, 002027 |
| 6-phosphogluconate dehydrogenase, decarboxylating | Pgd | 13 | 0, 709492257 | 0, 003216 |
| Glutathione S-transferase P 1 | Gstp1 | 3 | 0, 715568979 | 0, 006782 |
| Alanine—tRNA ligase, cytoplasmic | Aars | 9 | 0, 724055571 | 0, 00406 |
| T-complex protein 1 subunit epsilon | Cct5 | 7 | 0, 725355973 | 0, 008302 |
| Destrin | Dstn | 4 | 0, 726632332 | 0, 005658 |
| Ubiquitin-like modifier-activating enzyme 1 | Uba1 | 16 | 0, 727024557 | 0, 005177 |

(*Continued*)

| NC-MDSC vs B16-MDSC differentially expressed proteins | | | | |
|---|---|---|---|---|
| **Protein names** | **Gene names** | **Peptides** | **Fold change** | ***P* value** |
| ATPase Asna1 | Asna1 | 2 | 0, 72757609 | 0, 007588 |
| Ezrin | Ezr | 26 | 0, 730834893 | 0, 005634 |
| Phosphatidylethanolamine-binding protein 1 | Pebp1 | 3 | 0, 734400826 | 0, 005071 |
| Ribosomal RNA processing protein 1 homolog A | Rrp1 | 2 | 0, 738249657 | 0, 006636 |
| Transketolase | Tkt | 11 | 0, 741843927 | 0, 00586 |
| Peroxiredoxin-6 | Prdx6 | 8 | 0, 743801046 | 0, 002992 |
| Protein DJ-1 | Park7 | 5 | 0, 749901306 | 0, 007964 |
| Heat shock protein HSP 90-alpha | Hsp90aa1 | 23 | 0, 750975375 | 0, 003701 |
| Chloride intracellular channel protein 1 | Clic1 | 8 | 0, 751762163 | 0, 005866 |
| 40S ribosomal protein S18 | Rps18;Gm10260 | 4 | 0, 753299597 | 0, 005027 |
| Glutathione S-transferase Mu 1 | Gstm1 | 7 | 0, 753382783 | 0, 008871 |
| Ras suppressor protein 1 | Rsu1 | 2 | 0, 753677287 | 0, 004846 |
| Proteasome subunit alpha type-1 | Psma1 | 6 | 0, 761777735 | 0, 007526 |
| Bifunctional purine biosynthesis protein PURH | Atic | 9 | 0, 765684291 | 0, 00599 |
| Macrophage scavenger receptor types I and II | Msr1 | 6 | 1, 303825752 | 0, 009559 |
| Protein Sf3b2 | Sf3b2 | 8 | 1, 310630121 | 0, 004769 |
| Malate dehydrogenase, mitochondrial | Mdh2 | 10 | 1, 316433466 | 0, 005662 |
| ATP-dependent RNA helicase DDX42 | Ddx42 | 2 | 1, 326602896 | 0, 0084 |
| Beta-1, 4 N-acetylgalactosaminyltransferase 1 | B4galnt1 | 5 | 1, 328280926 | 0, 003965 |
| Legumain | Lgmn | 5 | 1, 363515033 | 0, 003449 |
| E3 UFM1-protein ligase 1 | Ufl1 | 3 | 1, 368089173 | 0, 005859 |
| Brain acid soluble protein 1 | Basp1 | 8 | 1, 369738842 | 0, 007428 |
| Cytochrome b-245 heavy chain | Cybb | 8 | 1, 379745693 | 0, 007504 |
| NADH dehydrogenase [ubiquinone] 1 beta subcomplex subunit 8, mitochondrial | Ndufb8 | 2 | 1, 379768717 | 0, 009965 |
| RNA-binding protein FUS | Fus | 4 | 1, 383579198 | 0, 003469 |
| Annexin A2 | Anxa2 | 23 | 1, 387238034 | 0, 001975 |
| Hexokinase-2 | Hk2 | 12 | 1, 391029042 | 0, 004338 |
| Protein Ahnak | Maoa | 82 | 1, 397110044 | 0, 003518 |

(*Continued*)

| NC-MDSC vs B16-MDSC differentially expressed proteins | | | | |
|---|---|---|---|---|
| **Protein names** | **Gene names** | **Peptides** | **Fold change** | ***P* value** |
| Succinate dehydrogenase [ubiquinone] flavoprotein subunit, mitochondrial | Ckb | 11 | 1, 400581193 | 0, 007454 |
| Chromodomain-helicase-DNA-binding protein 4 | Nampt | 5 | 1, 401800102 | 0, 004666 |
| Plasminogen receptor (KT) | Itpa | 3 | 1, 417180455 | 0, 003521 |
| Thyroid hormone receptor-associated protein 3 | Hnrnpl | 5 | 1, 418157791 | 0, 002434 |
| Zinc-binding alcohol dehydrogenase domain-containing protein 2 | Rnh1 | 5 | 1, 432808549 | 0, 002205 |
| Normal mucosa of esophagus-specific gene 1 protein | Bcap31 | 2 | 1, 447669214 | 0, 006718 |
| Pro-cathepsin H | Ndufs4 | 2 | 1, 448045875 | 0, 0024 |
| Annexin A4 | Atp6v1e1 | 20 | 1, 469858566 | 0, 004418 |
| Isopentenyl-diphosphate Delta-isomerase 1 | Hnrnpk;Gm7964 | 4 | 1, 484479927 | 0, 007223 |
| Heterogeneous nuclear ribonucleoprotein U-like protein 2 | Ldha | 7 | 1, 512224046 | 0, 003377 |
| Cytochrome b-c1 complex subunit Rieske, mitochondrial | Dhx15 | 6 | 1, 514801192 | 0, 000731 |
| RNA-binding protein 25 | Pgk1 | 3 | 1, 519285254 | 0, 005808 |
| Lysosome-associated membrane glycoprotein 1 | Pygl | 4 | 1, 528207246 | 0, 005381 |
| Dhx16 protein | Ndufv2 | 2 | 1, 535132714 | 0, 006593 |
| Probable ATP-dependent RNA helicase DDX6 | Ndufv1 | 2 | 1, 624024139 | 0, 006046 |
| Cell division cycle 5-like protein | Ndufaf2 | 2 | 1, 657520224 | 0, 006832 |
| Lysosome-associated membrane glycoprotein 2 | Tomm70a | 3 | 1, 684964459 | 0, 007944 |
| ADP/ATP translocase 1 | Rap1b;Rap1a | 5 | 1, 714237166 | 0, 009989 |
| Epoxide hydrolase 1 | Ptpn1 | 2 | 1, 800951684 | 0, 001541 |
| Metastasis-associated protein MTA1 | Ltf | 3 | 2, 002434335 | 0, 005013 |
| Arginase-1 | Vps33a | 10 | 2, 305826701 | 4, 13E-06 |
